# Supplementary material for: Predicted pH-dependent stability of SARS-CoV-2 spike protein trimer from interfacial acidic groups
Source: Comput Struct Biotechnol J. 2021 Sep 2;19:5140–8. doi: 10.1016/j.csbj.2021.08.049 (PMC8410215; doi:10.1016/j.csbj.2021.08.049)
Supplement: Supplementary data 1 [file mmc1.docx]

**Supp. Fig. 1.** Spike protein monomer burial within a trimer. Monomers are listed vertically according to the clustering derived for Fig. 1 (6zb4A and a group of locked forms at the top), and with sequence variation horizontally, referenced to the domain structure. A heat map of burial SASA (d-SASA) for monomer within trimer is shown, from zero (white) with increasing (brown) colour, capped at d-SASA = 80 Å^2^. A smaller vertical heat map gives d-SASA per monomer (white, smaller to purple, greater).

**Supp. Fig. 2.** Resolution of structures in the 6 sub-groups of S protein trimers. (a) A scatter plot of segment Manders’ overlap coefficients against reported resolution, for the 36 trimers of the (colour-coded) 6 sub-groups. A resolution threshold of 3.0 Å is marked. (b) Calculated contributions to the change of S trimer stability, from pH 7.5 to pH 5, are shown for various groups of residues. The plot is analogous to that shown in Fig. 4, but only for the locked and closed form sub-groups, and including only structures with resolution of 3.0 Å or better.

**Supp. Fig. 3.** Δp*K*_a_ values for different conformational forms, predicted with PROPKA. Predicted Δp*K*_a_s for ionisable groups (or subsets of ionisable groups) of interest are averaged for calculations over the 6 sub-groups of S protein trimers. For reference, the averages (for all sub-groups) of aspartic and glutamic acid sidechain, and histidine sidechain, Δp*K*_a_s are shown as dashed lines.

**Supp. Fig. 4.** Monomer burial within trimer for members of D614Gset. Variation in both S1 and S2 subunit contributions to d-SASA contribute to the large overall range of d-SASA values exhibited within D614Gset. RBD up or down monomer conformations are indicated.

**Supplementary Fig. S1**

**Supplementary Fig. S2**

**Supplementary Fig. S3**

**Supplementary Fig. S4**

**Supplementary Table 1.** Monomer PDB/chain identifiers are given in pairs with down or up states for the RBD. These 387 monomers from 129 spike protein trimers form the spike387 set. The order of monomers (left to right, top to bottom) is that given in the clustered heat maps of Fig. 1 and Supplementary Fig. S1.

| 6ZB4A | down | 7A96B | up | 7CWUC | up | 7CT5B | up | 7K8WG | down | 7JV6B | down | 7K8ZA | down | 7KECB | up |
| --- | --- | --- | --- | --- | --- | --- | --- | --- | --- | --- | --- | --- | --- | --- | --- |
| 6ZB4B | down | 7A96C | down | 7CWLB | up | 7K8SA | down | 6ZHDC | down | 7JV6E | down | 7KJ2A | down | 7KEAB | up |
| 6ZB4C | down | 7A93B | up | 7A94C | down | 7K8SB | down | 7K8UA | down | 7K8ZC | down | 7KJ5A | down | 7KDLB | up |
| 6ZB5A | down | 7DK3A | down | 6ZXNB | down | 7K8SC | down | 7JWBA | down | 7LCNA | down | 7KDJA | down | 7JWBC | up |
| 6ZB5B | down | 7DK3B | down | 6ZXNC | down | 7K8YB | down | 7K8WA | down | 7LCNC | down | 7KEBA | down | 6X2AB | up |
| 6ZB5C | down | 7DCCE | up | 7A25B | down | 7K8YD | up | 7JZNC | up | 7LCNK | down | 7KE9A | down | 6VSBB | down |
| 6XR8A | down | 7DCCI | up | 7A25C | down | 7K8YE | up | 6Z97A | down | 6X6PA | down | 6VYBA | down | 7A4NA | down |
| 6XR8B | down | 7DCCK | up | 7A29C | down | 7K8XA | down | 7CHHA | up | 6X6PB | down | 6X2AA | down | 7A4NB | down |
| 6XR8C | down | 7DD2C | up | 7DK3C | up | 7K8XB | down | 6ZHDA | up | 6X6PC | down | 7KECA | down | 7A4NC | down |
| 6ZGEA | down | 7DD2K | up | 6ZP0A | down | 7K8XC | up | 6Z43A | up | 6XM3C | down | 7KEAA | down | 6WPTB | up |
| 6ZGEB | down | 7DD8C | up | 6ZP0B | down | 7KKKA | down | 7CHHC | down | 7KDIA | down | 6XCMA | down | 7AD1C | down |
| 6ZGEC | down | 7DCXC | up | 6ZP0C | down | 7KKKC | down | 7K8UB | up | 7KDIB | down | 7KDLA | down | 7AD1A | down |
| 6ZGIA | down | 7DCXK | up | 6ZOYA | down | 7KKKE | down | 7JWBB | up | 7KDIC | down | 7KDHC | down | 6XF6B | up |
| 6ZGIB | down | 7DK6A | up | 6ZOYB | down | 7KKLA | down | 6XCNA | up | 7KDGA | down | 6X2BA | down | 7JWYC | down |
| 6ZGIC | down | 7DK6B | up | 6ZOYC | down | 7KKLC | down | 6XCNC | up | 7KDGB | down | 6X2AC | down | 6XLUC | down |
| 7JJIA | down | 7DK7A | up | 6ZP1A | down | 7KKLD | down | 6XCNE | up | 7KDGC | down | 7KJ3C | down | 6XM5A | down |
| 7JJIB | down | 7DK7B | up | 6ZP1B | down | 6ZDHA | up | 6XCMB | up | 7L06A | down | 7KJ2C | down | 7CN9C | down |
| 7JJIC | down | 7DK7C | up | 6ZP1C | down | 6ZDHB | up | 6XCMC | up | 7L06B | down | 7KJ5C | down | 6XM3B | up |
| 7DF3A | down | 7DK5A | up | 6ZOXA | down | 6ZDHC | up | 7JZLC | up | 7L06C | down | 7KDJC | down | 6XM4B | up |
| 7DF3B | down | 7DD2D | down | 6ZOXB | down | 6XF5A | down | 7K90A | down | 7KE6A | down | 7KEBC | down | 6ZOZA | down |
| 7DF3C | down | 7DD8D | down | 6ZOXC | down | 6XF5B | down | 7K90B | down | 7KE6B | down | 7KE9C | down | 6ZOZB | down |
| 7DDDA | down | 7DD8E | down | 7K43A | down | 6XF5C | down | 7K90C | down | 7KE6C | down | 6VYBC | down | 6ZOZC | down |
| 7DDDB | down | 7DCXD | down | 7K43B | down | 7K8TA | down | 6Z97B | up | 6X2CA | down | 7KECC | down | 7JWYB | down |
| 7DDDC | down | 7DK6C | down | 7K43E | down | 7K8TB | up | 7BYRB | down | 6X2CB | down | 7KEAC | down | 6XLUB | down |
| 6ZP2A | down | 7DK5B | down | 7CAIA | up | 7K8TC | down | 7KJ3A | up | 6X2CC | down | 7KDLC | down | 6XM5B | down |
| 6ZP2B | down | 7DK5C | down | 7CAIB | up | 7KL9B | up | 7KJ3B | up | 7KDKA | down | 6WPTC | up | 6XM0B | up |
| 6ZP2C | down | 7DK4A | up | 7CAIC | down | 7K4NA | up | 7KJ2B | up | 7KDKB | down | 6WPSA | down | 6XKLA | up |
| 6ZGHC | down | 7DK4B | up | 7CAKA | up | 7K4NB | up | 7KJ4A | up | 7KDKC | down | 6WPSB | down | 7AD1B | up |
| 6ZGHB | down | 7DK4C | down | 7CAKB | up | 7K4NE | up | 7KJ4B | up | 7KE4A | down | 6WPSE | down | 7C2LA | up |
| 7DDNB | down | 7KSGA | up | 7CAKC | up | 7KNEA | down | 7KJ4C | up | 7KE4B | down | 7KDHB | up | 7BYRA | up |
| 7DDNC | up | 7KSGB | up | 6XEYC | down | 7KNEC | down | 7KJ5B | up | 7KE4C | down | 6X79A | down | 6VSBA | up |
| 7DF4B | up | 6ZXNA | up | 6XEYA | down | 7KNHC | down | 7JZNB | up | 7L02A | down | 6X79B | down | 7CN9B | down |
| 7DF4D | down | 7CWUB | up | 7CWMA | down | 7KNBA | down | 6XM0C | down | 7L02B | down | 6X79C | down | 7CN9A | up |
| 7DF4C | down | 7CWSO | up | 7C2LB | down | 7KNBC | down | 6XM0A | down | 7L02C | down | 6XKLC | down | 6XS6C | up |
| 7DDNA | down | 7CWSQ | up | 7C2LC | down | 7KMZC | down | 6XM4A | down | 7L09A | down | 6WPTA | up | 6XS6A | up |
| 6ZGGB | up | 7CWSR | up | 6XEYB | down | 7KL9A | up | 6XM5C | down | 7L09B | down | 6XM4C | down | 6XS6B | up |
| 7A95C | up | 7CWUA | up | 7KNIA | up | 7KL9C | up | 6XM3A | down | 7L09C | down | 7JWYA | down | 6ZGHA | up |
| 6ZGGA | down | 7A25A | up | 7KNIB | up | 7JW0A | up | 7CHHB | down | 7KE8A | down | 6XLUA | down |  |  |
| 7A95A | up | 7A29A | up | 7KNIC | up | 7JW0B | up | 7JZNA | down | 7KE8B | down | 7BYRC | down |  |  |
| 6ZGGC | down | 7A29B | up | 7KNEB | up | 7JW0E | up | 7JZLA | down | 7KE8C | down | 6VSBC | down |  |  |
| 7A93A | up | 7CWLC | up | 7KNHA | up | 7JVCA | up | 7JZLB | up | 6X29A | down | 6XKLB | down |  |  |
| 7A93C | down | 7A94A | up | 7KNBB | up | 7JVCB | up | 7K8VC | down | 6X29B | down | 7K8WB | up |  |  |
| 7A95B | down | 7CWLA | down | 7KMZA | up | 7JVCE | up | 6ZWVA | down | 6X29C | down | 7LD1C | up |  |  |
| 7A97B | up | 7KSGC | up | 7KNHB | up | 7K8UC | up | 6ZWVB | down | 7KE7A | down | 6X2BB | up |  |  |
| 7A98A | up | 7CWMC | down | 7KMZB | up | 6Z43C | down | 6ZWVC | down | 7KE7B | down | 6X2BC | up |  |  |
| 7A98B | up | 7CWNA | up | 7KMSB | up | 6XF6C | down | 7K8VA | down | 7KE7C | down | 7KDJB | up |  |  |
| 7A98C | up | 7CWNC | up | 7KMSA | up | 6Z97C | down | 7K8VB | up | 6VXXA | down | 7KEBB | up |  |  |
| 7A97C | down | 7CWMB | up | 7KMSC | up | 6ZHDB | down | 7LD1A | up | 6VXXB | down | 7KE9B | up |  |  |
| 7A97A | up | 7A94B | down | 7CT5A | up | 6Z43B | down | 7LD1B | up | 6VXXC | down | 7K8ZB | up |  |  |
| 7A96A | up | 7CWNB | up | 7CT5C | up | 6XF6A | down | 7JV6A | down | 7KDHA | down | 6VYBB | up |  |  |

**Supplementary Table 2.** Monomer PDB/chain identifiers are given in pairs with down or up states for the RBD, S protein trimers in each of 6 sub-groups (labelled by the column headers).

| **locked** | **locked** | **pHlocked** | **pHlocked** | **diSlocked** | **diSlocked** | **D614Gset** | **D614Gset** | **closed** | **closed** | **open** | **open** |
| --- | --- | --- | --- | --- | --- | --- | --- | --- | --- | --- | --- |
| 6ZB4A | Down | 6XLUA | Down | 6ZOZA | Down | 7KDKA | Down | 6VXXA | Down | 7A98A | Up |
| 6ZB4B | Down | 6XLUB | Down | 6ZOZB | Down | 7KDKB | Down | 6VXXB | Down | 7A98B | Up |
| 6ZB4C | Down | 6XLUC | Down | 6ZOZC | Down | 7KDKC | Down | 6VXXC | Down | 7A98C | Up |
| 6ZB5A | Down | 6XM5A | Down |  |  | 7LWSA | Down | 6X6PA | Down | 7KJ4A | Up |
| 6ZB5B | Down | 6XM5B | Down |  |  | 7LWSB | Down | 6X6PB | Down | 7KJ4B | Up |
| 6ZB5C | Down | 6XM5C | Down |  |  | 7LWSC | Down | 6X6PC | Down | 7KJ4C | Up |
| 6XR8A | Down | 7JWYA | Down |  |  | 7KRQA | Down | 6X29A | Down | 7KNIA | Up |
| 6XR8B | Down | 7JWYB | Down |  |  | 7KRQB | Down | 6X29B | Down | 7KNIB | Up |
| 6XR8C | Down | 7JWYC | Down |  |  | 7KRQC | Down | 6X29C | Down | 7KNIC | Up |
| 6ZGEA | Down |  |  |  |  | 7KRRA | Up | 6X2CA | Down | 7K4NA | Up |
| 6ZGEB | Down |  |  |  |  | 7KRRB | Down | 6X2CB | Down | 7K4NB | Up |
| 6ZGEC | Down |  |  |  |  | 7KRRC | Down | 6X2CC | Down | 7K4NE | Up |
| 6ZGIA | Down |  |  |  |  | 7KRSA | Up | 6ZP0A | Down | 7KSGA | Up |
| 6ZGIB | Down |  |  |  |  | 7KRSB | Down | 6ZP0B | Down | 7KSGB | Up |
| 6ZGIC | Down |  |  |  |  | 7KRSC | Down | 6ZP0C | Down | 7KSGC | Up |
| 7JJIA | Down |  |  |  |  | 7BNMA | Down | 6ZOYA | Down | 7CWNA | Up |
| 7JJIB | Down |  |  |  |  | 7BNMB | Down | 6ZOYB | Down | 7CWNB | Up |
| 7JJIC | Down |  |  |  |  | 7BNMC | Down | 6ZOYC | Down | 7CWNC | Up |
| 7DDDA | Down |  |  |  |  | 7BNNA | Down | 6ZP1A | Down | 7KMSA | Up |
| 7DDDB | Down |  |  |  |  | 7BNNB | Up | 6ZP1B | Down | 7KMSB | Up |
| 7DDDC | Down |  |  |  |  | 7BNNC | Down | 6ZP1C | Down | 7KMSC | Up |
| 6ZP2A | Down |  |  |  |  | 7BNOA | Up | 6ZOXA | Down | 7LD1A | Up |
| 6ZP2B | Down |  |  |  |  | 7BNOB | Up | 6ZOXB | Down | 7LD1B | Up |
| 6ZP2C | Down |  |  |  |  | 7BNOC | Down | 6ZOXC | Down | 7LD1C | Up |
